# Selection of a protein solubilization method suitable for phytopathogenic bacteria: a proteomics approach

**DOI:** 10.1186/s12953-015-0062-9

**Published:** 2015-02-05

**Authors:** Carolina B Malafaia, Myrzânia L Guerra, Túlio D Silva, Patrícia MG Paiva, Elineide B Souza, Maria TS Correia, Márcia V Silva

**Affiliations:** Programa de Pós-Graduação em Ciências Biológicas - Centro de Ciências Biológicas - Universidade Federal de Pernambuco, Rua Prof. Nelson Chaves s/n, Cidade Universitária, CEP 50670-901 Recife, PE Brasil; Programa de Pós-Graduação em Fitopatologia - Universidade Federal Rural de Pernambuco, Av. Dom Manoel de Medeiros, s/n - Dois Irmãos, CEP: 52171-900 Recife, PE Brasil; Departamento de Bioquímica, Universidade Federal de Pernambuco, Rua Prof. Moraes Rego s/n, Cidade Universitária, 50670-420 Recife, PE Brasil; Departamento de Biologia, Universidade Federal Rural de Pernambuco, Av. Dom Manoel de Medeiros, s/n - Dois Irmãos, CEP: 52171-900 Recife, PE Brasil

**Keywords:** *Acidovorax citrulli*, *Pectobacterium carotovorum subsp. carotovorum*, *Ralstonia solanacearum*, Proteome analysis, Two-dimensional gel electrophoresis

## Abstract

**Background:**

Finding the best extraction method of proteins from lysed cells is the key step for detection and identification in all proteomics applications. These are important to complement the knowledge about the mechanisms of interaction between plants and phytopathogens causing major economic losses. To develop an optimized extraction protocol, strains of *Acidovorax citrulli*, *Pectobacterium carotovorum* subsp. *carotovorum* and *Ralstonia solanacearum* were used as representative cells in the study of phytopathogenic bacteria. This study aims to compare four different protein extraction methods, including: Trizol, Phenol, Centrifugation and Lysis in order to determine which are more suitable for proteomic studies using as parameters the quantity and quality of extracted proteins observed in two-dimensional gels.

**Results:**

The bacteria studied showed different results among the tested methods. The Lysis method was more efficient for *P. carotovorum* subsp. *carotovorum* and *R. solanacearum* phytobacteria, as well as simple and fast, while for *A. citrulli*, the Centrifugation method was the best. This evaluation is based on results obtained in polyacrylamide gels that presented a greater abundance of spots and clearer and more consistent strips as detected by two-dimensional gels.

**Conclusions:**

These results attest to the adequacy of these proteins extraction methods for proteomic studies.

## Background

The practice of agriculture brings as consequence the occurrence of plant diseases in levels that require their control. The most recommended method for this control is the use of genetic resistance [1]. However, not all plants are resistant to pathogens, and not every resistant variety is adapted to different regions of cultivation [2].

The bacteria *Acidovorax citrulli* (*Ac*), *Pectobacterium carotovorum* subsp. *carotovorum* (*Pcc*) and *Ralstonia solanacearum* (*Rs*), respectively cause bacterial fruit blotch, which is the main bacterial disease of melon culture, being responsible for heavy losses in production and depreciation of fruits [3]; soft rot in several hosts, among which lettuce, potatoes and tomatoes [4]; and bacterial wilt, which is the main worldwide vascular disease and attacks more than 50 botanical families, mostly the Solanaceae family, causing great economic losses [5,6].

The proteome is defined as the set of proteins expressed in a cell, tissue or any biological sample at a given time or under specific conditions [7]. The identification and characterization of these microorganisms using proteomic technologies can integrate the knowledge base necessary for the understanding of the mechanisms that phytobacteria use to cause diseases in their host [8]. In comparison with genomic studies, investigations at the proteome level provide detailed information, such as the abundance of proteins and post-transcriptional modifications [9,10].

The extraction of proteins is challenging, inconsistent and has been a problem for scientists [11]. Many techniques, including physical methods and those based on detergents, are available for protein extraction and are used for various purposes [12]. In proteomic studies, the development of an extraction method that can produce high yields and result in the complete dissolution, breakdown, denaturing and reduction of the greatest possible number of proteins present in the sample is an absolutely essential step for good results in two-dimensional gel electrophoresis (2D-PAGE) and mass spectrometry [13]. However, there are few studies that have compared the efficiency of these methods [14,15].

In this study, we compared four extraction methods: Trizol, Phenol, Centrifugation and Lysis, to determine their effectiveness in the separation of proteins by 2D-PAGE of three important phytobacteria: *Acidovorax citrulli*, *Pectobacterium carotovorum* subsp. *carotovorum* and *Ralstonia solanacearum*.

## Results

In this study, four different extraction methods were compared to determine which of them increase the protein solubilization of phytobacteria for subsequent analysis by 2D-PAGE. Considering that non-protein impurities can severely affect the quality of 2D-PAGE separation, this study was critical to evaluate, standardize and select efficient methods for protein analysis of *Acidovorax citrulli*, *Pectobacterium carotovorum* subsp*. carotovorum* and *Ralstonia solanacearum*.

The four extraction methods tested were effective in obtaining and concentrating proteins and the results are presented in Table 1. Although all methods presented appropriate yields for bacteria *Ac* and *Pcc*, the largest amount of proteins was obtained by the Centrifugation method. However, for *Rs* the best result was observed with the Lysis method, where there was a significant difference compared to the other methods tested.

**Table 1:** **The mean ± SD of protein concentrations (μg/μl) of all strains obtained by four different methods from mass of bacteria growth of 1 × 10** ^**7**^ **CFU/ml**

**Strains**	**Methods**			
	**Trizol**	**Phenol**	**Centrifugation**	**Lysis**
*Acidovorax citrulli*	8.83 ± 0.15	7.58 ± 0.17	11.34 ± 0.15	9.83 ± 0.18
*Pectobacterium carotovorum* subsp*. carotovorum*	8.90 ± 0.10	7.82 ± 0.08	10.54 ± 0.05	8.19 ± 0.18
*Ralstonia solanacearum*	8.74 ± 0.23	7.03 ± 0.13	8.61 ± 0.05	9.08 ± 0.00

**Figure 1:**
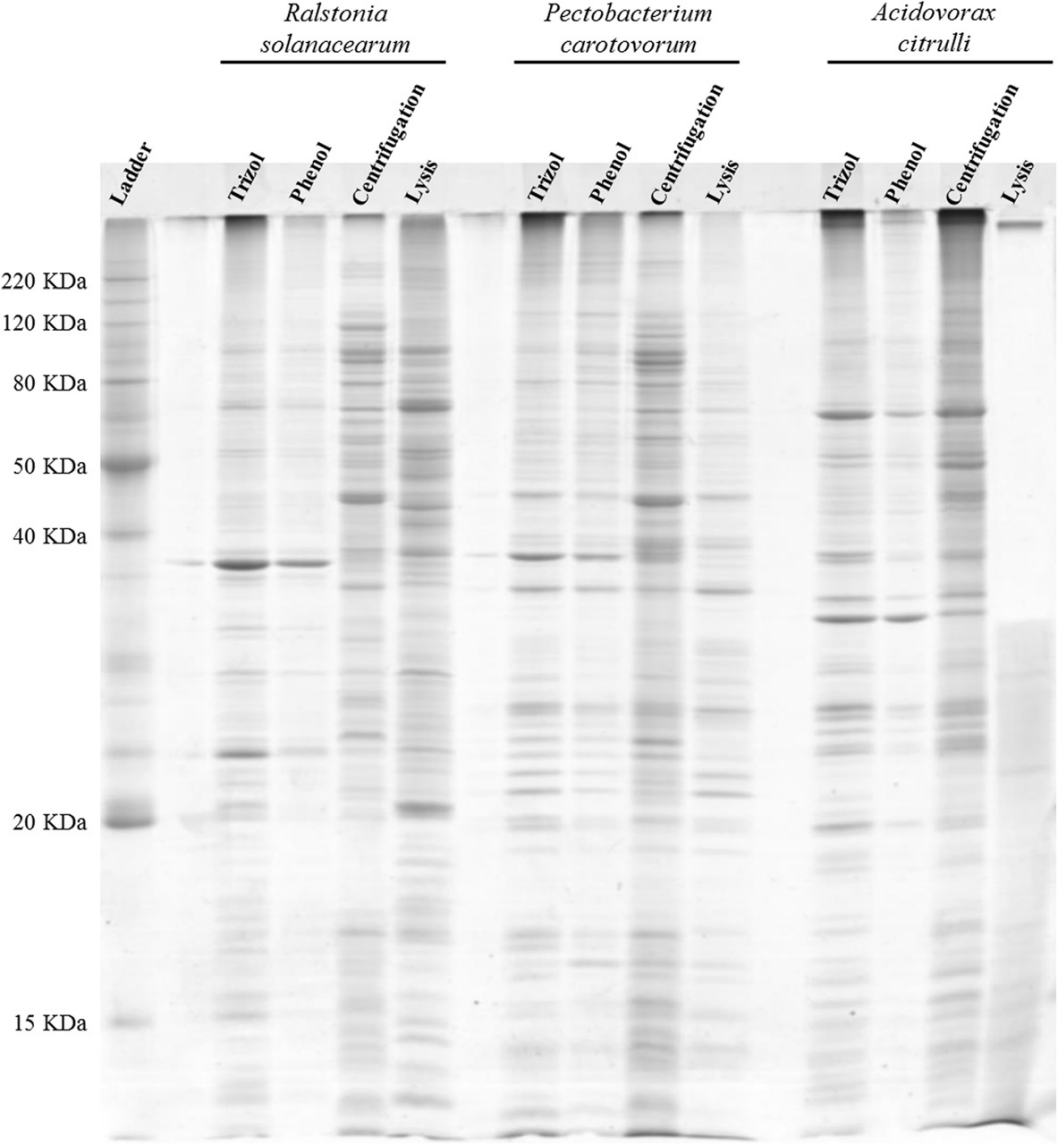
**SDS-PAGE of the different extraction methods in phytopathogenic bacteria.** SDS-PAGE stained with Coomassie brilliant blue illustrating the intracellular proteins of the phytobacteria *Ralstonia solanacearum*, *Pectobacterium carotovorum* subsp. *carotovorum*, and *Acidovorax citrulli* extracted by four different methods: Trizol, Phenol, Centrifugation and Lysis, Marker BenchMark™ Protein Ladder (Invitrogen) – KDa.

The SDS-PAGE analysis showed that the extractions presented good quality proteins, with well-defined bands without signs of degradation (Figure 1). For bacteria *Rs* and *Pcc* it was noted that all methods seem well suited. However, for *Ac* the Lysis method presented a loss of proteins with a molecular weight above 38KDa in addition to little definition of the bands, which from this analysis suggest that this method is less efficient compared to the other methods tested for this specie. In this gel, it is possible to observe that there is a difference in the patterns and intensity of the bands observed amongst the methods in each of the phytobacteria. Thus, for the study in question, the best extraction method was considered the one that is most comprehensive, namely, the method that presents the greatest possible number of proteins with the best definition in 2D-PAGE gels.

**Figure 2:**
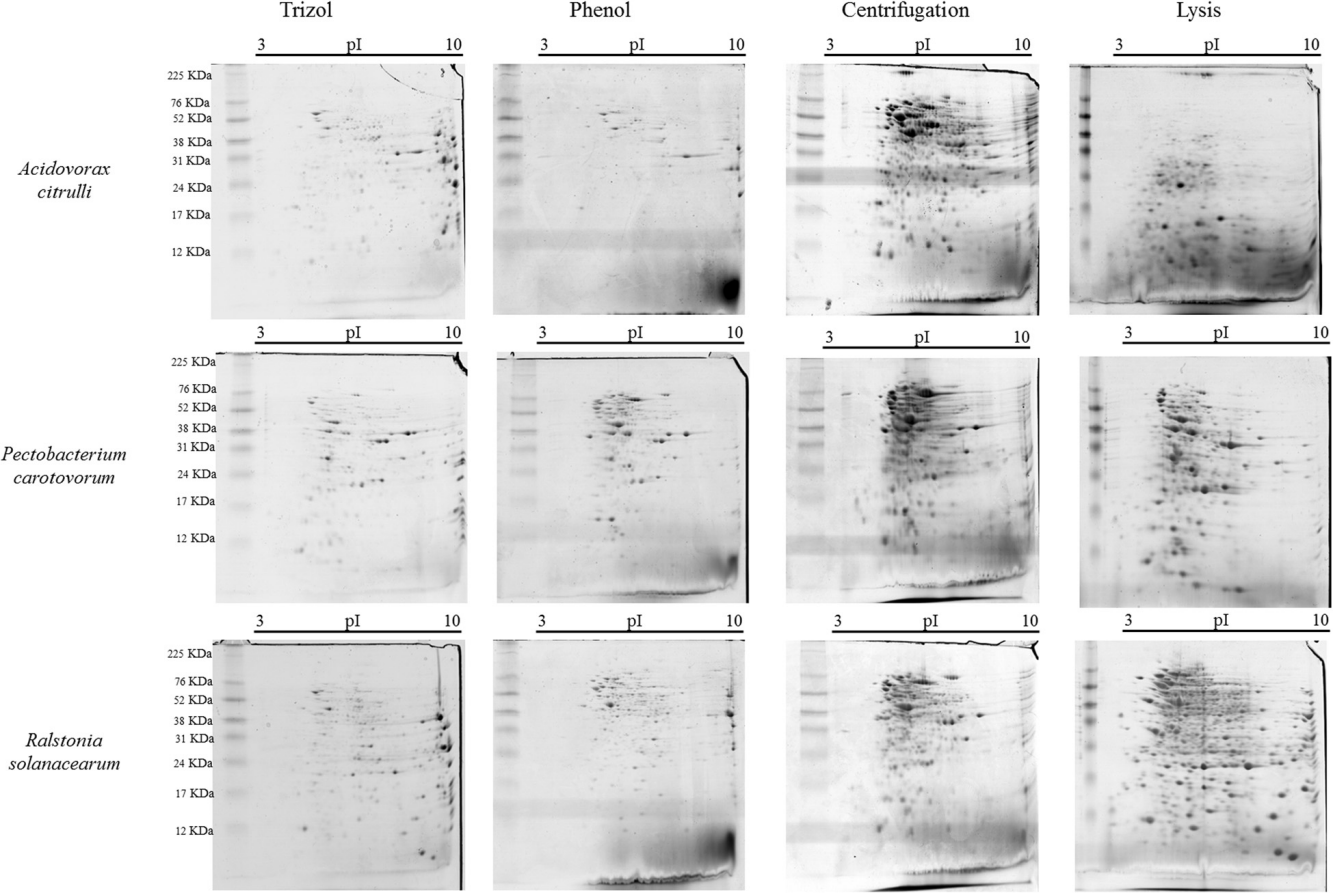
**Abundance of proteins in different extraction methods.** 2D-PAGE stained with Coomassie brilliant blue illustrating intracellular proteins, focused on 7 cm strips, pI 3–10, of the phytobacteria *Ralstonia solanacearum*, *Pectobacterium carotovorum* subsp. *carotovorum*, and *Acidovorax citrulli* extracted by four different methods: Trizol, Phenol, Centrifugation and Lysis, Marker Rainbow (GE Life Sciences).

The results of the two-dimensional gels (Figure 2 and Table 2) showed clarity and resolution, but were different for each of the bacteria. To define which method is best suited for the organism under study, one should consider the relative quality of the sample for analysis in 2D-PAGE and the number of protein spots obtained. *Ac* presented the best results with the Centrifugation method, showing 224 spot with a pH distribution between 4 to 7 and a molecular weight of 10 to 80 KDa; the bacteria *Pcc* and *Rs* presented respectively 212 spots, pH of 4 to 7 and molecular weight between 10 and 70 KDa and 369 spots, pH of 4 to 9 and a molecular weight of 20 to 70 KDa. These results showed a good range and are recommended for use in proteomic studies.

**Table 2:** **Number of spots of all strains obtained by the four different methods**

**Strains**	**Methods**			
	**Trizol**	**Phenol**	**Centrifugation**	**Lysis**
*Acidovorax citrulli*	164^a^ ± 5	43^b^ ± 4	224^c^ ± 8	126^d^ ± 4
*Pectobacterium carotovorum* subsp*. carotovorum*	152^a^ ± 3	151^a^ ±8	172^b^ ± 5	212^c^ ± 3
*Ralstonia solanacearum*	148^a^ ± 3	196^b^ ± 4	183^c^ ± 5	369^d^ ± 4

Superscript letters (a-d) indicate statistically significant relationships between methods (p <0.05).

## Discussion

Proteomic studies of high resolution depend mainly on a sample of good quality, so the method applied in the extraction of proteins is a key step to that end [11]. There is a great diversity of types of samples, therefore, an efficient protein isolation process for each one of them must be assumed empirically and tested in order to determine its real efficiency for the sample used, in order to obtain reproducible results in addition to the greatest possible representativeness of proteins in 2D gels [10]. During sample preparation in bacteria cells, problems may arise in cellular rupture due to the presence of thick cell walls and polysaccharide capsule in certain bacterial groups. Some bacteria can be lysed just by lysis buffer constituents, while others must be mechanically broken; in some cases it is necessary to use enzymes for the digestion of the cell wall [16].

Although many methods have been developed and reported, there is no single method for efficient isolation of all proteins of interest (cytosolic soluble or of a highly insoluble membrane) of an organism. Therefore, protein extraction methods continue to be a challenge for scientists in the accurate analysis of proteins [11]. In this regard, the chosen method should be simple and quick, with low cost and toxicity. These are important aspects in the selection of the method to be used, without selectively losing proteins while removing contaminants to the maximum extent possible [10].

The Lysis method has been applied in others organisms, such as sugarcane [17], soybean [11] and also in *Xanthomonas campestris* pv. *viticola* [18] with similar efficiency to that observed in this work. The lysis buffer composition allows quick access to the proteins, promoting denaturation, keeping them in the primary structure and thus protecting them against degradation agents. The preparing of protein samples consists in three fundamental steps, present in all methods: cell disruption, inactivation or removal of interfering and proteins solubilization [19].

The solubilization of proteins is considered the most problematic step in preparing protein samples for proteomic studies. The better solution is the buffer with a combination of urea and thiourea, associated with appropriate detergents, as tested by Chan and collaborators [20] for *Prorocentrum triestinum*. They observed an increase in the number of spots in electrophoresis gels when using urea and an even greater increase when using the combination of urea and thiourea. This is due to the fact that urea is a chaotropic agent, efficient in the rupture of hydrogen bonds, denaturing proteins by breaking the non-covalent and ionic links between aminoacid residues [19], leading to the split and denaturation of proteins. In turn, thiourea is very suitable for breaking hydrophobic interactions, increasing the solubilization of membrane proteins [21,22]. CHAPS and DTT are two important components in the proteins solubilization because they prevent hydrophobic interactions and promote the re-oxidation of disulfide bonds, respectively, avoiding the loss of proteins by aggregation or precipitation.

There are several advantages to the use of the Lysis method in protein extraction: it is a method that is simple, fast (about 1 h), most interfering materials (non-protein components) are effectively removed, the proteins are protected against degradation by proteases, thus not requiring the addition of protease inhibitors, in addition to having low toxicity. Furthermore, the composition of the extraction buffer ensures that proteins are under the same conditions as 2D-PAGE.

The Centrifugation method was very efficient for *Acidovorax citrulli*, result that suggests its utilization for specific studies with this specie. The presence of SDS in the extraction buffer used in this method allows access to the proteins by breaking the membrane and, associated with heating at 100°C, inactivating proteases. The use of DNAse I and RNAse A enzymes, with subsequent precipitation with acetone, guarantees the elimination of contaminating in the final sample [23].

Some studies have focused on comparing protocols for protein extraction from a wide variety of organisms. For example, a study of lactic acid bacteria, which presented the comparison of three extraction methods for sonication, Centrifugation and FastPrep, found the best results with the latter [24]. In aphids, the TCA/acetone-based method was the more efficient in comparisons for 2DE than detergent and phenol based methods [13]. Unlike the results obtained in this work, in dinoflagellates the Trizol method presented better results when compared to the Lysis method [25]. Proteomic studies conducted with the bacterial phytopathogen *Xanthomonas axonopodis* pv. *citri* showed that the Phenol method was employed with success [26]. In some cases, it is necessary to develop a new method due to peculiarities of the sample in question as noted by Barbarino and Lourenço in 2005 [27] due to high concentrations of salts present in the sample.

## Conclusions

For new proteomic studies with organisms that have not been registered in the literature, a pre-test of different methods for the preparation of the sample is strongly recommended in order to determine which is best suited for this type of analysis. In the case of the phytobacteria used in this study, the recommended methods are Centrifugation for *Acidovorax citrulli* and Lysis for *Pectobacterium carotovorum* subsp. *carotovorum* and *Ralstonia solanacearum*.

## Methods

### Growing conditions

Bacterial isolates were *Acidovorax citrulli* (*Aac* 1.12), *Pectobacterium carotovorum* subsp. *carotovorum* (*Pcc* 31), and *Ralstonia solanacearum* (*Rs* CGH 26), obtained by Culture Collection of the Phytobacteriology Laboratory of the Agronomic Department of Universidade Federal Rural de Pernambuco, Brazil. Were grown in 20 ml NYD medium (dextrose 10 g/l; meat extract 3 g/l; yeast extract 5 g/l; peptone 5 g/l) during 24 h at 28°C under constant agitation of 150 rpm for the formation of the pre-inoculate. Following this, 180 ml of the same media was added and the culture maintained under the same growth conditions for 24 h. Consequently, the bacterial growth was collected by centrifugation at 10.000 × g for 5 min, to obtain the cell mass for the extraction of total protein. Three biological replicas were made (independent cultures) and the samples were collected at an optical density (OD_600_ = 0.5 ± 0.05) corresponding to the exponential phase 1 x 10^7^ CFU/ml of each of the strains.

### Extraction of proteins

Four different protein extraction methods were tested including modified Trizol, Phenol, Centrifugation and Lysis. After extraction, the supernatants containing the proteins of each of the methods were stored at – 20°C until later analysis.

### Trizol method

Protein extraction followed the instructions set out by the manufacturer of Trizol (Invitrogen ®) with some modifications. Briefly, 500 μl Trizol reagent were added to the cell pellet and lyse cells in sample by pipetting up and down several times. Subsequently, 200 μl of chloroform were added to the cell lysate before shaking vigorously for 15 s. The mixture was allowed to stand for 5 min at 25°C before being centrifuged at 12000 × g for 15 min at 4°C. The aqueous phase was removed. 300 μl of ethanol were added in order to resuspend the reddish bottom layer and the mixture centrifuged at 8000 × g for 5 min at 4°C. Supernatant was transferred to a new tube and 1.5 ml of isopropanol were added. The mixture was allowed to stand for at least 20 min for protein precipitation at 25°C. It was then centrifuged at 12000 × g for 10 min at 4°C. The pellet obtained was briefly washed with 95% ethanol before allowed to air dry. Finally, the proteins were ressolubilized in 500 μl of sample preparation solution (7 M Urea; 2 M thiourea; 4% CHAPS).

### Phenol method

Total protein extraction was done as described by Metha and Rosato in 2001 [28]. The cell pellets were washed in phosphate buffer (7 mM K_2_HPO_4_; 3 mM KH_2_PO_4_; 0.15 mM NaCl; pH 7.2) and 750 μl of extraction buffer were added (0.7 M sucrose; 0.5 M Tris–HCl; 30 mM HCl; 50 mM EDTA; 0.1 M KCl and 40 mM DTT; pH 8.5), followed by incubation for 15 min (25°C). The same volume of phenol was added, and after 15 min of agitation in a vortex, the suspension was centrifuged at 14.000 x g for 3 min at 4°C and the phenolic phase was recovered. This procedure was repeated two more times. Proteins were precipitated with the addition of 5 volumes of 0.1 M ammonium acetate in methanol. The precipitate was washed with 1 ml of 80% acetone and solubilized as previously described.

### Centrifugation method

The pellets were resuspended in 500 μl of extraction buffer (0.3% SDS; 200 mM DTT; 48 mM Tris; 28 mM HCl; pH 8.8). The microcentrifuge tubes containing the cell suspension were agitated gently for 10 min at 4°C, followed by removal of the cells by centrifugation at 14.000 x g for 10 min at 4°C. The extraction was incubated at 100°C for 5 min and then cooled on ice. Subsequently, 24 μl of assay buffer (0.5 M Tris; 476 mM HCl; 50 mM MgCl_2_ pH 8.5; 1 mg/ml DNAse I; 0.25 mg/ml RNAse A) were added and the extraction incubated again for 15 min on ice. The reaction was stopped by the addition of four volumes of acetone cooled on ice and precipitation of proteins was left to occur for 20 min on ice. The cellular debris were removed by centrifugation at 14.000 × g for 10 min at 4°C [29]. The proteins were resolubilized in in 500 μl of sample preparation solution (7 M Urea; 2 M thiourea; 4% CHAPS)

### Lysis method

The centrifuged pellets of bacteria were resuspended in 500 μl of lysis buffer (7 M urea; 2 M thiourea; 4% CHAPS) and homogenized in a vortex for 5 min at 25°C. The homogenized sample was centrifuged at 10.000 x g for 30 min at 4°C. The supernatant was transferred to a new 1.5 ml tube [17].

### Quantification of proteins

Total cellular protein concentrations were determined using a commercial protein colorimetric assay kit, 2D Quant Kit, according to the manufacturer’s protocol (GE Life Sciences®) with bovine serum albumin (BSA) as a standard of measurement and absorbance at 480 nm. The kit is reported to not interfere with any chemicals used during extraction protocols and is therefore compatible with isoelectric focusing (IEF). The samples and the standard were read in triplicate.

### SDS-PAGE

100 μg of protein were applied in a 15% acrylamide separating gel was used with stacking at 4% for SDS-PAGE on SE 600 Ruby Standard Dual Cooled Vertical Unit. The acrylamide gel was run at 40 mA for 15 min and then at 100 mA for 2 h (Electrophoresis Power Supply – EPS 601 - GE Life Sciences) [30] in SDS buffer (124 mM Tris; 960 mM glycine; 17.5 mM SDS). 10 μl of protein molecular weight standard BenchMark™ Protein Ladder (Invitrogen) were used. At the end of electrophoresis, the gels were visualized by staining with Coomassie brilliant blue (5% acetic acid; 20% methanol; 0.2% Comassie Brilliant Blue R-250) and then decolorized with 0.5% acetic acid and 20% methanol.

### 2D-PAGE

Two-dimensional electrophoresis (2-DE) was carried out according to the method of Görg and collaborators [31]. In the first dimension isoelectric focusing, 100 μg proteins were added to a rehydration buffer (7 M urea; 2 M thiourea; 2% CHAPS; 2 mM DTT; 1% IPG buffer pH 3–10 and 0.2% bromophenol blue) for a final volume of 250 μl. The sample was loaded onto 13 cm pH 3-10NL immobiline DryStrips (GE Life Sciences) with overnight rehydration, followed by isoelectric focusing for a total of 15.500 V/hrs. Strips were equilibrated in SDS equilibration buffer (6 M urea, 30% glycerol, 2% SDS) for 15 min with 10 mg/ml DTT, then 15 min in fresh buffer with 25 mg/ml 15 min. The second dimension was performed in homogeneous vertical acrylamide gel 15%. The equilibrated strips were applied onto the gel and sealed with agarose 0.5% and bromophenol blue 0.01%. The proteins electrophoretic separation was performed at 15°C in two stages: the first at 15 mA per fixed gel for 20 min and the second at 45 mA per gel for approximately 2 hours. Rainbow was used the molecular weight standard (GE Life Sciences). After the second dimension electrophoresis, the proteins were stained as in the SDS-PAGE.

Rainbow was used as the molecular weight standard (GE Life Sciences). After the second dimension electrophoresis, the proteins were stained as in the SDS-PAGE.

### Analysis of two-dimensional gels

After stained, the gels were scanned using an ImageScanner (Amershan Biosciences) scanner in transparency mode with a resolution of 300 dpi and images were saved in .mel format. These were analyzed using the ImageMaster Platinum v. 7.0 (Amershan Biosciences) computer program. The detection of each spot of protein was validated by manual inspection. The program provided the number of spots of each of the gels.

### Statistical analysis

Data were analyzed by oneway analysis of variance (ANOVA) followed by Tukey’s posthoc test and shown as mean and standard deviation. In all statistical analyses, p < 0.05 as taken as the level of significance.

## Abbreviations

*Ac*: *Acidovorax citrulli*
*Pcc*: *Pectobacterium carotovorum* subsp. *carotovorum*
*Rs*: *Ralstonia solanacearum*
2D-PAGE: Two-dimensional polyacrylamide gel electrophoresis
SDS-PAGE: Sodium dodecyl sulfate - polyacrylamide gel electrophoresis
2DE: Two dimensional electrophoresis
TCA: Trichloroacetic acid
NYD: Nutrient yeast dextrose
OD_600_: Optical density 600 nm
CHAPS: 3-[(3-Cholamidopropyl)dimethylammonio]-1-propanesulfonate
SDS: Sodium dodecyl sulfate
DTT: Dithiothreitol
BSA: Bovine serum albumin
IEF: Isoelectric focusing
